# CircDDX17 inhibits invasive progression of pituitary adenomas by sponging miR-1279 and regulating CADM2 expression

**DOI:** 10.3389/fonc.2023.1268644

**Published:** 2023-11-01

**Authors:** Xiao Yue, Fengming Lan, Weiping Liu

**Affiliations:** ^1^ Department of Neurosurgery, Xijing Hospital, Air Force Medical University, Xi’an, Shaanxi, China; ^2^ National Cancer Center/National Clinical Research Center for Cancer/Cancer Hospital and Shenzhen Hospital, Chinese Academy of Medical Sciences and Peking Union Medical College, Shenzhen, China

**Keywords:** pituitary adenoma, invasion, circRNA, circDDX17, miR-1279, CADM2

## Abstract

**Purpose:**

Increasing evidence has revealed that circDDX17 plays significant regulatory roles in tumor progression. In the present study, we investigated the role of circDDX17 in pituitary adenomas (PAs).

**Methods:**

Reverse transcription–quantitative PCR was performed to detect the expression of Circular RNA DDX17 (circDDX17), microRNA-1279 (miR-1279), and cell adhesion molecule 2 (CADM2) in PA tissues. Cell abilities of migration and invasion were examined by wound healing and transwell assays. Dual-luciferase reporter, RNA immunoprecipitation, and RNA pull-down assays were applied to confirm the associations among circDDX17, miR-1279, and CADM2. Xenograft tumor experiments were performed to investigate the roles of circDDX17 *in vivo*.

**Results:**

In the present study, we found that circDDX17 was downregulated in PA tissues correlated with invasion, tumor size, and progression-free survival of patients with PA. Enforced expression of circDDX17 significantly inhibited migration and invasion through miR-1279. Notably, CADM2 was verified as the direct binding target of miR-1279, and silencing the expression of CADM2 reverses the tumor suppressing effects induced by circDDX17 overexpression. We demonstrated that circDDX17 upregulated the expression of CADM2 by sponging miR-1279, which suppressed the invasive biological behaviors of PA.

**Conclusion:**

CircDDX17 may serve as a tumor suppressor and potential promising biomarker and effectively therapeutic target for the management of PA.

## Introduction

Pituitary adenomas (PAs), which account for 10%–25% of the intracranial tumors, are the most common classification of pituitary disorder (1). There have been limited dramatic advances in the diagnosis and treatment of PAs in recent years (2). Therefore, it is critical and urgent to discover the molecular regulations and potential targets for better clinical management of PA.

Circular RNA (circRNA) is a unique-particular category of non-coding RNA, in general, generated from the back-splicing mechanism of exons of pre-mRNAs. The majority of circRNAs exhibit a stable and conserved nucleotide base sequence throughout evolution across various tissues and species (3). CircRNAs have been considered as functional molecules to regulate transcriptional control and modulate splicing translation in different biological and pathophysiological backgrounds (4). Emerging evidences demonstrate that miRNAs can specifically interact with circRNAs, serving as miRNA sponge to influence clinical behavior, impact cellular immune responses, and directly disturb the pathogenic mechanisms such as proliferation, invasion, apoptosis, and cell cycle (5). Multiple documents have highlighted the significance of circRNA involvement in shaping both the characteristic features observed in clinical cancer cases as well as their contribution to functionally activating physiological or pathological processes through modulation of diverse gene expressions (6). Nevertheless, the biological functions and regulatory mechanisms of circRNAs in PA are reported minimally and remain elusive.

CircDDX17 is downregulated and acts as a tumor suppressor in many cancers, such as colorectal cancer, breast cancer, and prostate cancer (7). CircDDX17 enhances coxsackievirus B3 replication through regulating miR-1248/NOTCH receptor 2 axis (7). CircDDX17 acts as a competing endogenous RNA for miR-605 in breast cancer progression (8). CircDDX17 reduces 5-fluorouracil resistance and hinders tumorigenesis in colorectal cancer by regulating miR-31-5p/KANK1 axis (9). In this paper, we select circDDX17 as a targeted gene to discuss its regulatory roles and potential applications as a future biomarker or therapeutic implication by sponging miR-1279 in PA.

## Materials and methods

### Patients and clinical samples

A total of 76 patients diagnosed with primary PA in The First Affiliated Hospital (Xijing Hospital) of the Air Force Medical University between June 2006 and May 2010 were recruited in this study. The inclusion criteria were as following: (1) the primary tumor, (2) both radiological and pathological characteristics diagnosed with PA, and (3) no treatment of chemotherapy or radiotherapy. Frozen tissue specimens of PA were subjected to RNA examining after operation. This research was approved by the ethics committee of The First Affiliated Hospital (Xijing Hospital) of the Air Force Medical University. Informed consent was obtained from all the enrolled patients prior to recruitment. All tissue specimens were conducted and made anonymous by complying with the ethical and legal standards. The collected clinical samples were first flash-frozen in liquid nitrogen and then stored at −80°C refrigerator before examination.

### Cell culture and transfection

HP75 cell line was obtained from the Institute of Biochemistry and Cell Biology, Chinese Academic of Sciences. Isolation of cells for primary culture of PA tissue was performed as described previously (10). Cells were cultured in high-glucose Dulbecco’s modified Eagle medium (DMEM; 11995040, Gibco, USA) supplemented with 10% fetal bovine serum (10099141C, Gibco, USA) in humidified incubator containing 5% CO_2_ at 37°C. si-circDDX17 (5′-CCACAAATTTGGAGCAAGA-3′, knock-down circDDX17), si-circNC (5′-TTCTCCGAACGTGTCACGT-3′, negative control), si-CADM2 (5′-TACGTCCAAGGTCGGGCAGGAAGA-3′, knock-down CADM2), and si-NC (5′-CGTACGCGGAATACTTCGA-3′, negative control) were synthesized from GenePharma, Shanghai, China. To construct a circDDX17-overexpressing plasmid (circDDX17), we synthesized circDDX17 cDNA and cloned it into the pcD-ciR vector (Geneseed Biotech Co., Guangzhou, China). An empty plasmid served as the negative control (vector). All synthetic compounds were transfected by Lipofectamine 2000 (Invitrogen, Carlsbad, CA, USA).

### Subcellular localization

Cytoplasmic and nuclear fractions from PA cells were isolated using the Cytoplasmic & Nuclear RNA Purification Kit (Norgen Biotek, Thorold, Canada).

### Reverse transcription–quantitative PCR

Reverse transcription–quantitative PCR (RT-qPCR) was performed by SYBR Green PCR mixture (Invitrogen) with a gene-specific primer/probe mix and DNA template. Reaction mixtures were incubated at 95°C for 5 min, followed by 40 cycles of 95°C for 10 s, 60°C for 30 s, and 72°C for 1 s. Signals were detected at the end of each cycle. SDS 2.4 software (Applied Biosystems) was performed to calculate the cycle threshold (Ct) values. The 2^−ΔΔCT^ method was used to calculate relative expression. **Additional File 1**, **Table S1** lists the specific primers.

### Luciferase assays

The genomics sequences including the wild-type (WT) binding site or the mutated (MUT) binding site were all cloned into the firefly luciferase reporter vector. The associated and regulated relationship of circDDX17 and miR-1279 was verified by dual-luciferase reporter assay (Promega, Madison, WI, USA). Another luciferase reporter assay was applied to investigate the targeting relationship of miR-1279 and CADM2. PA cells were co-transfected with WT or MUT luciferase reporter vector. After co-transfection, a dual-luciferase reporter system was applied to measure the luciferase activity following 48 h of incubation.

### Western blot

Total protein lysates were separated by sodium dodecyl sulfate polyacrylamide gel electrophoresis and transferred to polyvinylidene difluoride membranes (Millipore, Billerica, MA, USA). Primary antibodies detecting targeted proteins were added in the blot and incubated for 12 h. Glyceraldehyde 3-phosphate dehydrogenase (GAPDH) was used as control. Finally, the combined signals were detected by Chemistar™ High-sig ECL Western Blot Substrate (Tanon, Shanghai, China). The antibodies CADM2 (1:1,000, AB_2854219), Matrix metalloproteinase 2 (MMP2) (1:1,000, AB_2792342), MMP9 (1:1,000, AB_2809982), GAPDH (1:1,000, AB_2107310), and Horseradish Peroxidase (HRP)-conjugated Immunoglobulin G (IgG) anti-rabbit (1:10,000, AB_228341) were commercially acquired from Thermo Fisher Scientific (Waltham, MA, USA).

### RNA pull-down assay

Lipofectamine™ 2000 was applied to transfect biotinylated control or circRNA probe into PA cells. RNA pull-down assay was performed by using the Magnetic RNA-Protein Pull-Down Kit (Thermo Fisher Scientific, Waltham, MA, USA) according to the manufacturer’s protocol. The recruited mRNA was following examined by RT-qPCR analysis.

### RNA immunoprecipitation assay

RNA immunoprecipitation (RIP) was performed by an RNA Binding Protein Immunoprecipitation kit purchased from Millipore. PA cells were lysed using RIP lysis buffer. Cell lysates were centrifuged at 15,000 × g for 20 min at 4°C, and the supernatant was incubated with magnetic beads coated with 10 μg of control IgG or antibody overnight at 4°C, followed by digestion with proteinase K buffer. The magnetic beads were precipitated by a magnetic bar and then washed three times with the washing buffer. At the last, TRIzol reagent was applied to extract the immunoprecipitation RNA examined by RT-qPCR analysis.

### Wound healing assay

Approximately 4 × 10^5^ PA cells were seeded in six-well plates for wound healing assays. Then, a 200-μl pipette tip was applied to make a straight scratch. Every plate was washed with PBS more than three times and replaced with serum-free medium. Images of wounds were captured at the appropriate time to estimate the area occupied by migratory cells.

### Transwell assay

Transwell assay (Costar, New York, NY, USA) was performed to examine the invasion and migration capacities of PA cells *in vitro* according to the manufacturer’s protocol. Cells (4 × 10^5^) were collected and re-suspended in serum-free DMEM and then inoculated in the upper chamber, and 10% serum-containing medium was added to the lower chamber. In the migration assay, un-coated upper chambers were directly utilized for further analysis. The transwell was precoated with Matrigel (BD Biosciences) for cell invasion determination. After 24 h, the invaded or migrated cells were fixed with 4% paraformaldehyde for 10 min and 0.5% stained crystal violet for 20 min at room temperature. Finally, the filter membrane was photographed and counted in three random fields.

### Mouse xenografts

All animal experiment procedures were carried out according to the regulations and internal biosafety and bioethics guidelines of The First Affiliated Hospital (Xijing Hospital) of the Air Force Medical University. All applicable international, national, and/or institutional guidelines for the care and use of animals were followed. Six- to eight-week-old female BALB/c nude mice (Vital River, Beijing, China) were used *in vivo* detection. Thirty mice were randomly divided into two groups: vector and circDDX17. Transfected HP75 cells (5 × 10^6^ per mice) were injected into the right flanks of mice. After 28 days observation, the mice were performed euthanasia by CO_2_, and subcutaneous tumors were extracted for further experiments.

### Statistical analysis

SPSS version 13.0 (SPSS, Chicago, IL, USA) was applied to assess and analyze statistical data. Paired two-tailed Student’s t-test or Chi-square test was performed to analyze the significance between groups. The Pearson’s correlation coefficient analysis was performed to analyze the correlationships. Log-rank test was used to discover the significance, and Kaplan–Meier curves were for survival analysis. Statistical significance was determined as P < 0.05 (*) or 0.01 (**).

## Results

### CircDDX17 was downregulated in PA tissues and correlated with poor clinical prognosis

**Figure 1:**
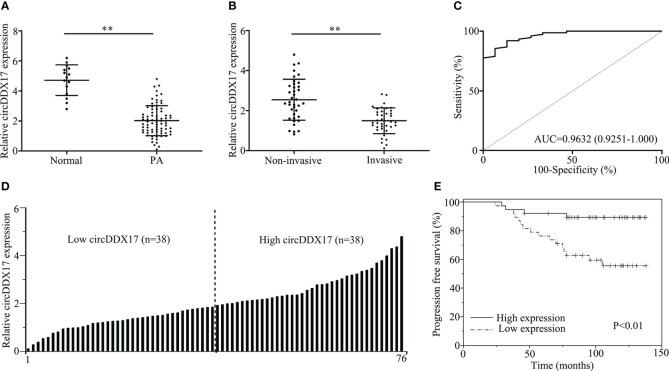
CircDDX17 was downregulated in pituitary adenoma (PA) tissues and correlated with poor clinical prognosis. **(A)** The detection of circDDX17 between PA tissues (n = 76) and normal pituitary tissues (n = 15) by RT-qPCR. **(B)** RT-qPCR analysis of the circDDX17 expression in PA samples between invasive (n = 38) and non-invasive (n = 38) patients. **(C)** ROC curve analysis based on circDDX17 levels for distinguishing patients with PA from normal controls. **(D)** Two groups were divided according to the levels of circDDX17. **(E)** Analysis of progression-free survival (PFS) using Kaplan–Meier survival curves. **p < 0.01.

To identify the circRNAs involved in the occurrence and progression of PA, RT-qPCR was initially applied to detect the relative expression levels of circDDX17 as demonstrated in **Figure 1**. Analysis of the expression circDDX17 in patients with PA (n = 76) revealed a significant difference in contrast to healthy controls (n = 15) after normalization (p < 0.01; **Figure 1A**). Further experiments confirmed a decreasing tendency of circDDX17 in the invasive group compared with the non-invasive group (p < 0.01; **Figure 1B**). In the subsequent analysis, we conducted and analyzed the associations between clinic-pathological characteristics and levels of circDDX17 expression. The significantly statistical differences of circDDX17 expression were observed among different Knop stages, tumor size, and recurrent status; however, no obvious differences were found in age and gender (**Table 1**). To evaluate the accuracy of circDDX17 levels for the diagnosis of patients with PA, Receiver Operating Characteristic Curve (ROC) curve analysis and the corresponding area under curve (AUC) values were calculated to discriminate PA from the general population. ROC analyses exhibited that circDDX17 levels could act as a valuable biomarker to distinguish patients with PA from healthy controls, with AUC value of 0.9632 (95% CI, 0.9251–1.000) (**Figure 1C**). Subsequently, to analyze the effect of circDDX17 expression to the prognosis of patient with PA, all recruited patients were divided into two groups: low circDDX17 group (n = 38) and high circDDX17 group (n = 38) according to the median value of circDDX17 expression (**Figure 1D**). Survival analysis demonstrated a low level expression of circDDX17 correlated with poor progression-free survival (PFS) in contrast to the high expression of circDDX17 (**Figure 1E**).

**Table 1:** Association of circDDX17 expression in pituitary adenoma with different clinicopathological features.

Characteristic	Total no.	circDDX17 expression		P
		Low (n, %)	Low (n, %)	
Total No.	76	38	38	
Age (years)^+^				
≤44	32	15 (46.9)	17 (53.1)	NS
>44	44	23 (52.3)	21 (47.7)	
Gender				
Male	30	17 (56.7)	13 (43.3)	NS
Female	46	21 (45.7)	25 (54.3)	
Knosp				
0–II	38	11 (28.9)	27 (71.1)	<0.01
III–IV	38	27 (71.1)	11 (28.9)	
Tumor size (cm)				
<1 cm	16	0 (0.0)	16 (100.0)	<0.01
1–3 cm	31	10 (32.3)	21 (67.7)	
>3cm	29	28 (96.6)	1 (3.4)	
Recurrence				
Yes	20	19 (95.0)	1 (5.0)	<0.01
No	56	19 (33.9)	37 (66.1)	

^+^Median age was 44 years.

### Aberrant expressions of circDDX17 affect the migration and invasion of PA cell lines

**Figure 2:**
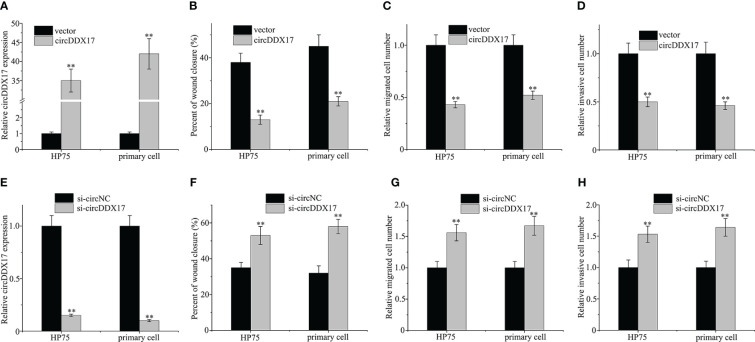
Aberrant expressions of circDDX17 affect the migration and invasion of PA cell lines. **(A)** Relative expression of circDDX17 when transfected with circDDX17 or control vector. **(B–D)** circDDX17 overexpression significantly reduced invasive and migrating capacity. **(E)** Downregulation of circDDX17 when transfected with si-circDDX17. **(F–H)** si-circDDX17 significantly increased invasive and migrating capacity. **p < 0.01.

The above results have suggested that circDDX17 was downregulated in PA tissues and correlated with a poor clinical prognosis; next, we performed a series of experimental assays to explore the function of circDDX17 in PA progression. We transiently transfected circDDX17 vector to increase its expression and explore the changes of biological functions in HP75 cells and primary PA cells (**Figure 2A**). As depicted in **Figure 2B**, upregulation of circDDX17 significantly suppressed cell migration analyzed by wound healing assay. Increasing circDDX17 effectively restrained the migrated ability of PA cells compared with control vector group (**Figure 2C**). Consistently, Matrigel invasion assays revealed that the invasive abilities of PA cells were also inhibited by overexpression of circDDX17 (**Figure 2D**). Moreover, si-circDDX17 was transfected into HP75 cells and primary PA cells to knockdown circDDX17 expression (**Figure 2E**). Knockdown of circDDX17 expression obviously increased the migration and invasion capabilities in contrast to si-circNC control group in PA cells as consistent with the circDDX17 overexpression tendency (**Figures 2F–H**). Together, these results indicated that circDDX17 could significantly inhibit PA cells invasive and migrating capacity.

### CircDDX17 acts as a sponge for miR-1279 in PA cells

**Figure 3:**
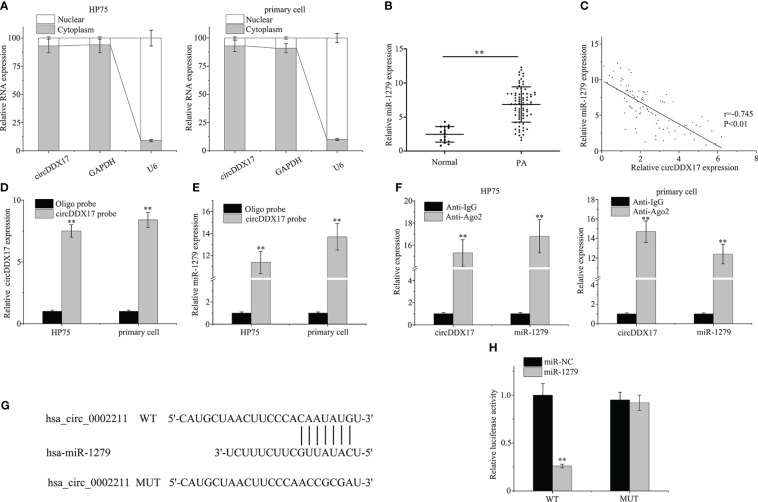
CircDDX17 acts as a sponge for miR-1279 in PA cell lines. **(A)** RT-qPCR analysis of circDDX17 in the cytoplasm and nucleus. **(B)** RT-qPCR was used to detect the miR-1279 expression level in normal samples (n = 15) and PA tissues (n = 76). **(C)** Pearson’s correlation analysis showed a negative correlation between circDDX17 and miR-1279 in patients with PA (r = −0.745, p < 0.01). **(D, E)** The relationship between miR-1279 and circDDX17 was verified by RNA pull-down assay. **(F)** RNA immunoprecipitation RT-qPCR analysis was performed in PA cells. **(G)** The putative binding sites between circDDX17 and miR-1279. **(H)** Dual-luciferase reporter assays were performed to confirm the association between circDDX17 and miR-1279. **p < 0.01.

As demonstrated in **Figure 3A**, circDDX17 was predominantly localized in the cytoplasm, occasionally in the nuclei of HP75 cells and primary PA cells. As circRNAs were reported as molecular sponges of miRNAs to regulate expression of targeted miRNAs, the publicly available “circBank” and “circInteractome” databases tools were employed to predict potentially possible binding miRNA candidates of circDDX17. miR-1279 was predicted by both circRNA database tools and chosen to further verification. Next, the expression levels of miR-1279 were examined in PA specimens by RT-qPCR, and the association of circDDX17 and miR-1279 was assessed. As depicted in **Figure 3B**, the expression of miR-1279 in PA tissues was obviously increased compared with the normal tissues. Pearson’s correlation coefficient analysis confirmed a significant negative correlation between the expression of circDDX17 and miR-1279 in PA tissues (r = −0.745, p < 0.01; **Figure 3C**). The biotin-labeled probe pull-down assay was implemented to investigate the directly binding interactions between circDDX17 and miR-1279, and the results revealed abundant enrichment of miR-1279 when treated with circDDX17 probe in contrast to the control oligo probe (**Figures 3D, E**). Furthermore, AGO2 RIP assay also demonstrated that AGO2-containing complex could accumulate both circDDX17 and miR-1279 in large amount compared with IgG-control (**Figure 3F**). Subsequently, the putative binding sites between circDDX17 and miR-1279 were predicted to further confirm the sponging function (**Figure 3G**). Dual-luciferase reporter assay demonstrated that the presence of miR-1279 mimic significantly inhibited the luciferase activity of the WT circDDX17 reporter, whereas no obvious change in the MUT reporter (**Figure 3H**). Collectively, the series findings proved that circDDX17 as a specific miRNA sponge could negatively regulate and directly bind to miR-1279 in PA.

### CircDDX17 regulates PA cells’ migratory and invasive abilities through sponging miR-1279

**Figure 4:**
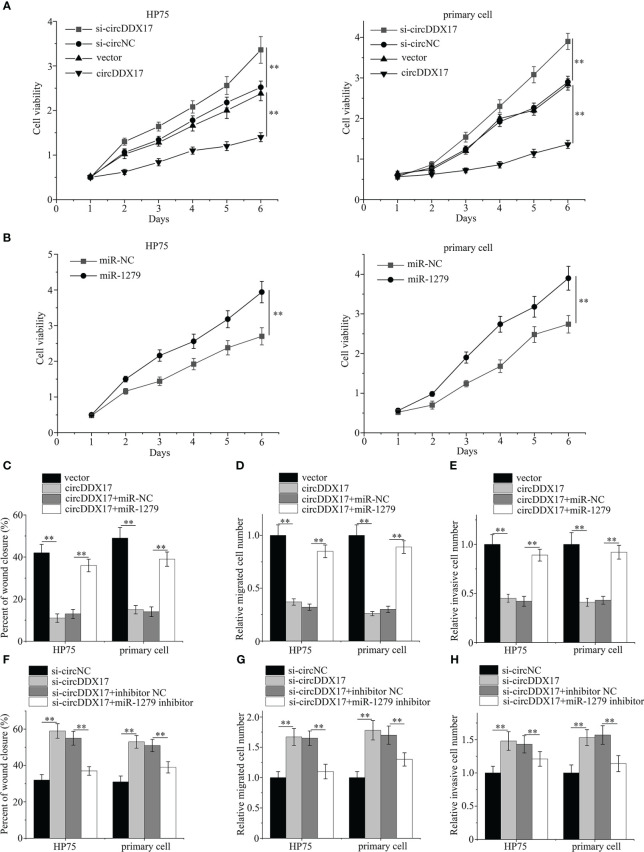
CircDDX17 regulates the migratory and invasive abilities in PA cells through sponging miR-1279. **(A)** Cell Counting Kit-8 proliferation assay in PA cells subjected to different circDDX17 treatments. **(B)** Cell Counting Kit-8 proliferation assay with overexpression miR-1279. **(C–E)** Cell migration and invasion assays in PA cells subjected to different treatments (vector, circDDX17 vector, circDDX17 vector + miR-NC, or circDDX17 vector + miR-1279). **(F–H)** Cell migration and invasion assays in PA cells subjected to different treatments (si-circNC, si-circDDX17, si-circDDX17 + NC inhibitor, or si-circDDX17 + miR-1279 inhibitor). **p < 0.01.

As shown in **Figure 4A**, upregulating the expressions of circDDX17 significantly inhibited the proliferation of PA cells, whereas decreasing circDDX17 levels promoted the cell proliferation compared with the corresponding control group. Overexpressed miR-1279 enhanced the proliferative ability when transfected with miR-1279 mimics (**Figure 4B**). In addition, rescued experiments were designed to confirm the inhibiting effects of circDDX17 through sponging miR-1279 and examined the aggressive biology by co-transfecting circDDX17 vector and miR-1279 mimics in HP75 cells and primary PA cells. Remarkably, upregulated miR-1279 levels could partially restrain the tumor-suppressing effects of circDDX17 in the migratory and invasive abilities (**Figures 4C–E**). Moreover, knockdown of circDDX17 expression obviously increased the migration and invasion capabilities, and the promoting effects were also alleviated by miR-1279 inhibitors (**Figures 4F–H**). Consistently, these results suggested that circDDX17 regulated PA cells’ migratory and invasive abilities through sponging miR-1279.

### CircDDX17 interfered the expression of CADM2

**Figure 5:**
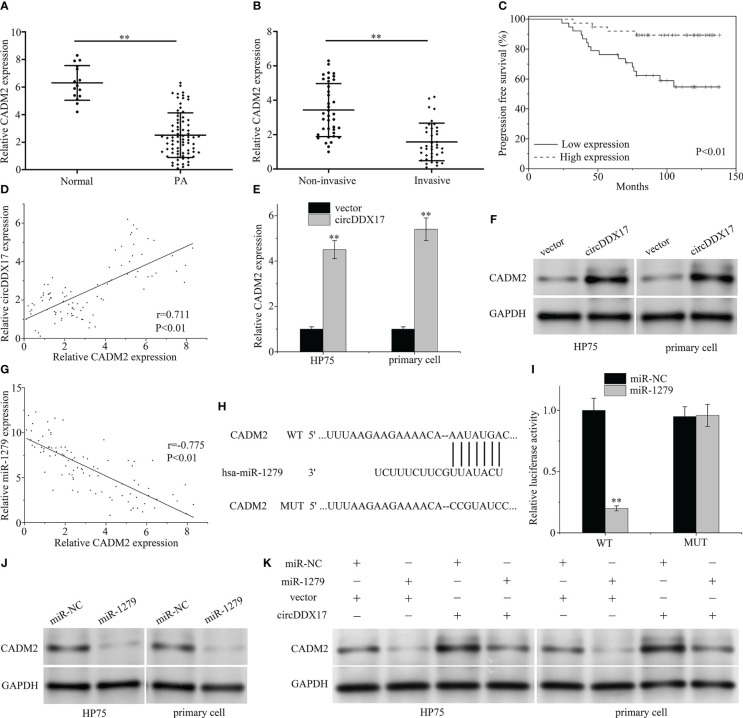
CircDDX17 interfered the expression of CADM2 through miR-1279. **(A)** RT-qPCR was used to detect the CADM2 expression level in normal pituitary tissues (n = 15) and PA tissues (n = 76). **(B)** RT-qPCR analysis of the CADM2 expression in PA samples between invasive (n = 38) and non-invasive (n = 38) patients. **(C)** Analysis of progression-free survival (PFS) using Kaplan–Meier survival curves. **(D)** Pearson’s correlation analysis revealed a positive correlation between circDDX17 and miR-1279 (r = 0.711, p < 0.01). **(E, F)** RT-qPCR and Western blot were used to detect the expression level of CADM2 when transfected with circDDX17 vector. **(G)** A negative correlation between circDDX17 and miR-1279 expression (r = −0.775, p < 0.01). **(H)** Sequence alignment between miR-1279 and the 3′UTR of CADM2. **(I)** pGL3-WT-ROR1-3’UTR-Luc and pGL3-MUT-ROR1-3′UTR-Luc reporters were transfected with miR-1279 mimics. **(J)** Western blot was used to detect the expression level of CADM2 when transfected with miR-1279 mimics. **(K)** CADM2 expression levels in different groups of PA cells were detected by Western blotting. **p < 0.01.

To explore the underlying mechanism of miR-1279 and predict the downstream targets, web-based software program, TargetScan, was applied and CADM2 searched as a candidate gene. In the beginning, the expression levels of CADM2 were examined in PA specimens by RT-qPCR, and the association of circDDX17 and CADM2 was evaluated. As demonstrated in **Figures 5A, B**, the expression of CADM2 in PA tissues was obviously reduced compared with the normal tissues and decreased even much more in the invasive group. Survival analysis demonstrated a low level expression of CADM2 correlated with poor survival, and, next, Pearson’s correlation coefficient analysis was applied to confirm a significant positive correlation between the expression of circDDX17 and CADM2 in PA tissues (r = 0.711, p < 0.01; **Figures 5C, D**). Upregulation of circDDX17 levels significantly increases the mRNA and protein expression (**Figures 5E, F**). Next, we investigated whether circDDX17 interfered the expression of CADM2 through regulating intermediate factor miR-1279. The correlation analysis displayed inverse association between miR-1279 and CADM2 (r = −0.775, p < 0.01; **Figure 5G**). As TargetScan calculated, the 3′ Untranslated Region (3′UTR) of CADM2 contained the putative binding sites of miR-1279 (**Figure 5H**). In addition, dual-luciferase reporter assay and Western blot confirmed the negative regulation and directly binding correlation between miR-1279 and CADM2 (**Figures 5I, J**). Moreover, upregulated circDDX17 also caused a significantly increasing on the expression level of CADM2 protein, whereas miR-1279 partly reversed the enrich containing of CADM2 (**Figure 5K**). Together, these data demonstrated that circDDX17 suppressed the invasive progression by eliminating miR-1279 and then interfering CADM2 expression.

### Silencing the expression of CADM2 reverses the invasive ability induced by circDDX17 overexpression

**Figure 6:**
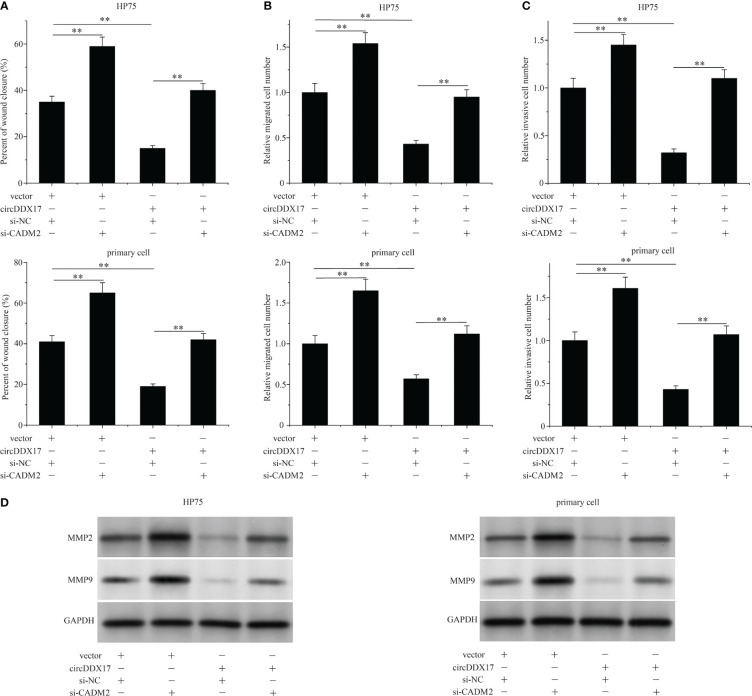
Silencing the expression of CADM2 reverses the invasive ability induced by circDDX17 overexpression. **(A–C)** Cell migration and invasion assays in PA cells in different groups (vector + si-NC, vector + si-CADM2, circDDX17 + si-NC, or circDDX17 + si-CADM2). **(D)** Western blot was used to detect the expression level of MMP2/9. **p < 0.01.

In this section, we were aimed to explore whether circDDX17 participated in the biologically invasive prognosis by forming circDDX17/miR-1279/CADM2 regulating axis and ultimately affecting the activity of CADM2. We co-transfected circDDX17 vector and CADM2 siRNA to explore the changes of biological functions in HP75 cells and primary PA cells. As expected, declined CADM2 containing significantly attenuated the migration suppressing effects of circDDX17 enrichment in PA cells analyzed by wound healing assay (**Figure 6A**). Analogously, increasing circDDX17 effectively inhibited the migrated and invasive abilities of PA cells in the previous results, whereas CADM2 downregulation also overrode this suppression effects (**Figures 6B, C**). Furthermore, increasing circDDX17 levels inhibited the expression of MMP2 and MMP9 levels, which was also reversed by CADM2 knockdown (**Figure 6D**). Collectively, these data indicated that the significant effects of circDDX17 in invasive ability were mediated by the activity of CADM2 expression via sponging miR-1279.

### CircDDX17 suppresses the growth of subcutaneous tumor established by PA cells *in vivo*

**Figure 7:**
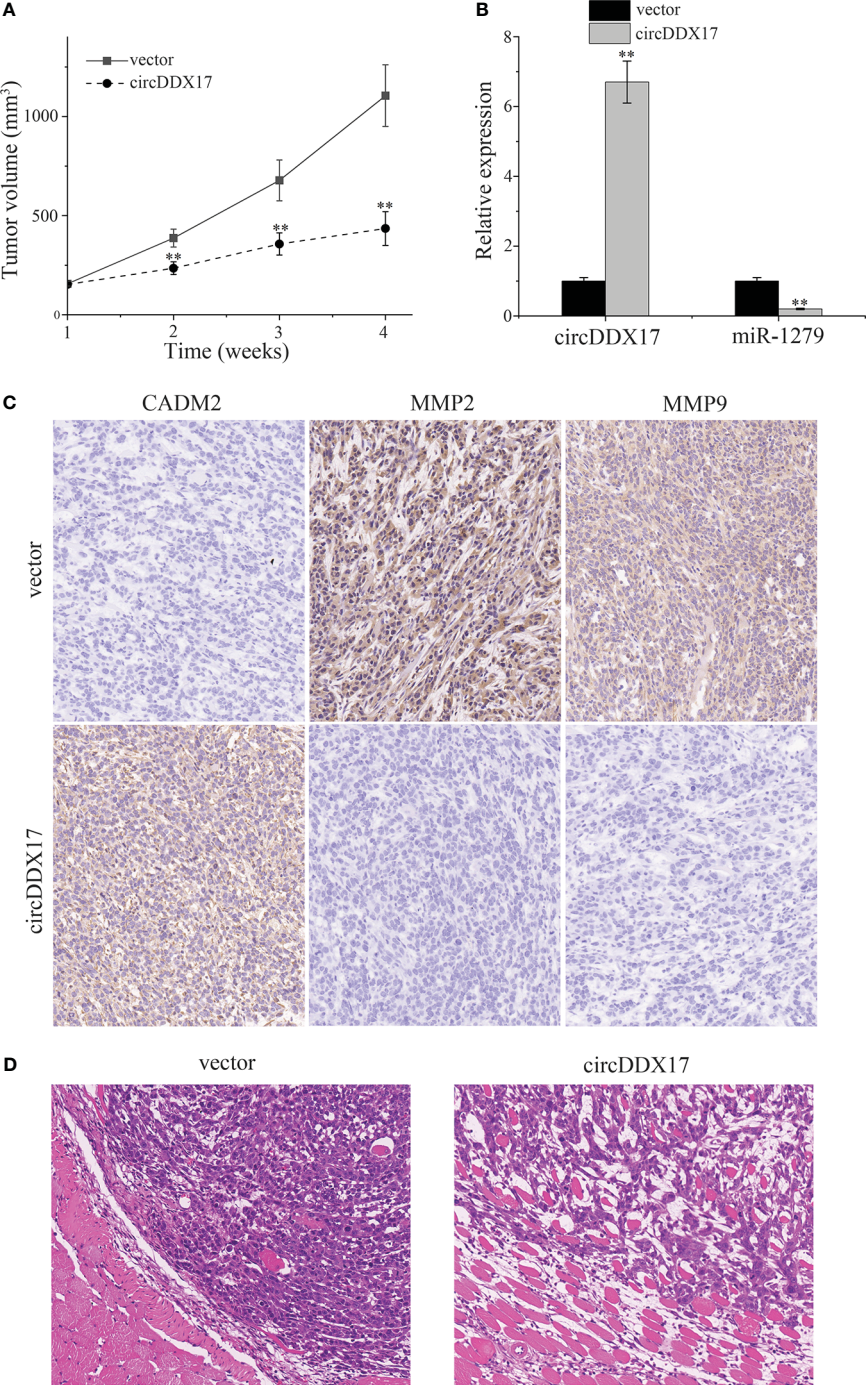
CircDDX17 suppresses the growth of subcutaneous tumor established by PA cells *in vivo*. **(A)** Subcutaneous tumors bearing HP75 cells were established and measured weekly. **(B)** RT-qPCR analysis of the expression level of circDDX17 and miR-1279 in the xenografts of nude mice. **(C)** Immunohistochemistry was performed to detect the expression of CADM2 and MMP2/9 in the xenografts of nude mice. **(D)** Hematoxylin and eosin staining in nude mice of the vector and circDDX17 groups. **p < 0.01.

HP75 cells were used to establish subcutaneous tumors for the further experiment *in vivo*. Xenograft growth assay indicated that overexpression circDDX17 could significantly inhibit tumor growth compared with the control groups from the second week (**Figure 7A**). RT-qPCR was used to examine solid tumors, and the levels of circDDX17 were upregulated, whereas the levels of miR-1279 were downregulated in the group of transfected with circDDX17 (**Figure 7B**). In addition, immunohistochemical analysis was performed to examine the downstream proteins. CircDDX17 overexpression significantly increased the downstream target of CADM2 expression, whereas migration-related proteins MMP2/9 were inhibited (**Figure 7C**). In the previous study, we have demonstrated that the levels of MMP2/9 were correlated with high invasive ability to cavernous sinus in PA (11). In addition, hematoxylin and eosin staining indicated that overexpression of circDDX17 could inhibit invasion of PA cells *in vivo* (**Figure 7D**). In conclusion, these data supported that overexpression of circDDX17 efficiently inhibits the growth and invasion of PA bearing subcutaneous tumors *in vivo*.

## Discussion

Although PAs are typically characterized as benign tumors, there are about 40% of patients with PA exhibiting invasive behaviors and resulting in poor prognosis. Nowadays, techniques and modern management of PA have achieved great improvement and provided best available treatment to patients (12). However, exploring the tumor molecular regulating mechanisms could improve the global judgments and recognize the genetics biology for developing future therapies.

Similar to other cancers, multiple risk genes for PA have been reported in the previous documents. CHST7 is a novel pituitary gland specific protein in SF-1 lineage adenomas with a potential role in gonadotroph cell proliferation and lineage differentiation in PA (13). Programmed cell death 10 (PDCD10) can induce aggressive behaviors of PAs by promoting cellular proliferation, migration, invasion, and epithelial-mesenchymal transition (EMT) through CXC motif chemokine receptor 2 (CXCR2)-AKT/extracellular regulated protein kinase (ERK) signaling axis (14). Genome-wide DNA methylation differences in non-functioning PA are correlated with postsurgical progression (15). Transcription factor general transcription factor IIB (GTF2B) could regulate aryl hydrocarbon receptor-interacting protein (AIP) protein expression and influence tumor phenotypes in growth hormone-secreting PA (16). Long non-coding RNA BBOX1 antisense RNA 1 (LncRNA BBOX1-AS1) promotes PA progression via sponging miR-361-3p to upregulate E2F1 expression (17). ACT001, a novel compound with effective anticancer activity, induces autophagic cell death (ACD) of PA cells via activating c-Jun N-terminal kinase (JNK) and P38 phosphorylation by binding with MEK4 (18). miR-149 regulates proliferation, migration, and invasion of PA cells by targeting a disintegrin and metalloproteinase 12 (ADAM12) and MMP14 (19).

With the advancements of sequencing technologies, an increasing number of circRNAs have been discovered. As an essential regulatory factor, circRNAs play a significantly pathophysiological role in the occurrence and progression of various disease by interfering many biological processes such as proliferation, invasion, migration, and differentiation (20). Currently, circRNAs are considered as potentially promising biomarkers and future targets for molecular-based therapy in all kinds of diseases, especially cancers (21). The molecular mechanisms and biology biological functions of circRNAs mainly focus on gene regulations, miRNA sponges, and protein transcriptions. As reported, circBCAR3 accelerates esophageal cancer tumorigenesis and metastasis via sponging miR-27a-3p (22). Aberrant nuclear export of Circular RNA NCOR1 (circNCOR1) underlies small mothers against decapentaplegic 7 (SMAD7)-mediated lymph node metastasis of bladder cancer (23). CircEZH2/miR-133b/insulin like growth factor 2 mRNA binding protein 2 (IGF2BP2) aggravates colorectal cancer progression via enhancing the stability of m6A-modified CREB1 mRNA (24). CircGPR137B/miR-4739/FTO feedback loop suppresses tumorigenesis and metastasis of hepatocellular carcinoma (25). CircSTX6 promotes pancreatic ductal adenocarcinoma progression by sponging miR-449b-5p and interacting with CUL2 (26). Circ_0017109 promotes lung tumor progression via activation of Wnt/β-catenin signaling due to modulating miR-671-5p/FZD4 axis (27). Hypoxia-induced exosomal circPDK1 promotes pancreatic cancer glycolysis via c-myc activation by modulating miR-628-3p/BPTF axis and degrading BIN1 (28).

Although dysregulations of circRNAs were frequently reported in various human cancers, there is currently few insightful understandings in PA. In the present study, we are committed to reveal the biology functions and molecular mechanisms of circDDX17 in PA. Initially, we demonstrated the decreasing tendency of circDDX17 in PA specimens, especially the invasive group. Furthermore, circDDX17 used as a valuable and novel biomarker in the diagnosis and prognosis of PA. Next, we performed a series of experimental assays to explore the function of circDDX17 in PA progression. The results indicated that circDDX17 functioned as an essential tumor suppressor in PA cells through inhibiting invasive and migrating capacity. To explore the probable mechanisms, circDDX17 as a specific miRNA sponge could negatively regulate and directly bind to miR-1279 in PA. More important, circDDX17 regulated the migratory and invasive abilities and exerted a tumor suppressing role in PA cells through sponging miR-1279, and upregulated miR-1279 levels could partially restrain the tumor-suppressing effects of circDDX17. Notably, CADM2 was verified as the direct binding target of miR-1279, and circDDX17 suppressed the invasive progression by eliminating miR-1279 and then interfering CADM2 expression. CADM2 as a number of cell adhesion molecule family has appeared among the top associations in a wide range of multiple cancers and plays an essential role in maintenance of cell polarity and normal cellular processes. CADM2 reported as a tumor inhibiting gene could suppress the Akt signaling pathway (29, 30). Moreover, silencing the expression of CADM2 reverses the tumor suppressing effects induced by circDDX17 overexpression. At last, we demonstrated the anti-tumor effects of circDDX17 in invasive ability were mediated by the activity of CADM2 expression via sponging miR-1279 *in vitro* and *in vivo*.

CircRNA-miRNA regulatory networks are involved in the tumor occurring biology and the activation of signaling pathway; hence, there is an urgent need to elucidate the molecular mechanisms of circRNAs and explore the associated options to achieve the success in cancer treatment (31). In conclusion, this study provides emerging evidences that circDDX17 as a novel tumor suppressing was downregulated and correlated with the invasion and prognosis in PA tissue. Furthermore, upregulation of circDDX17 could significantly suppress cell proliferation and invasion *in vitro* and *in vivo* by targeting the miR-1279/CADM2/MMP2/9 axis, suggesting circDDX17 as a potentially promising biomarker and effectively therapeutic target for the management of PA disease.

## Data availability statement

The raw data supporting the conclusions of this article will be made available by the authors, without undue reservation.

## Ethics statement

This study was performed in line with the principles of the Declaration of Helsinki. Approval was granted by the Ethics Committee of the Air Force Medical University (no. 81627806). All animal experiments were approved by the Animal Management Rule of the Chinese Ministry of Health (document 55, 2001) and were conducted in accordance with the approved guidelines and experimental protocols of the Air Force Medical University. Informed consent was obtained from all individual participants included in the study.

## Author contributions

XY: Data curation, Formal Analysis, Investigation, Writing – original draft. FL: Conceptualization, Data curation, Formal Analysis, Investigation, Writing – original draft. WL: Conceptualization, Funding acquisition, Methodology, Project administration, Resources, Supervision, Writing – original draft.
