# Supplementary material for: CircDDX17 inhibits invasive progression of pituitary adenomas by sponging miR-1279 and regulating CADM2 expression
Source: Front Oncol. 2023 Nov 1;13:1268644. doi: 10.3389/fonc.2023.1268644 (PMC10646483; doi:10.3389/fonc.2023.1268644)
Supplement: Supplementary file 1 [file Table_1.docx]

Table S1. Primer sequences for qRT-PCR

| Name | Forward (5’-3’) | Reverse (5’-3’) |
| --- | --- | --- |
| circDDX17 | TGCCAACCACAACATCCTCCA | CGCTCCCCAGGATTACCAAAT |
| miR-1279 | CTCAACTGGTGTCGTGGAGTCGGCAATTCAGTTGAGAGAAAGAA | CTCAACTGGTGTCGTGGAGTCGGCAATTCAGTTGAGAGAAAGAA |
| CADM2 | TCTATTCCAACAAGTCAGAAAATAATG | CGCTTAGACTTGATTTTGACGG |
| GAPDH | GGAGCGAGATCCCTCCAAAAT | GGCTGTTGTCATACTTCTCATGG |
| U6 | CGCTTCGGCAGCACATATAC | TTCACGAATTTGCGTGTCAT |
